# Ablation of peri-insult generated granule cells after epilepsy onset halts disease progression

**DOI:** 10.1038/s41598-017-18237-6

**Published:** 2017-12-21

**Authors:** Bethany E. Hosford, Shane Rowley, John P. Liska, Steve C. Danzer

**Affiliations:** 1 Department of Anesthesia, Cincinnati Children’s Hospital Medical Center, Cincinnati, OH 45229 USA; 2 Departments of Anesthesia and Pediatrics, University of Cincinnati, Cincinnati, OH 45267 USA; 3 Neuroscience Graduate Program, University of Cincinnati, Cincinnati, OH 45267 USA

## Abstract

Aberrant integration of newborn hippocampal granule cells is hypothesized to contribute to the development of temporal lobe epilepsy. To test this hypothesis, we used a diphtheria toxin receptor expression system to selectively ablate these cells from the epileptic mouse brain. Epileptogenesis was initiated using the pilocarpine status epilepticus model in male and female mice. Continuous EEG monitoring was begun 2–3 months after pilocarpine treatment. Four weeks into the EEG recording period, at a time when spontaneous seizures were frequent, mice were treated with diphtheria toxin to ablate peri-insult generated newborn granule cells, which were born in the weeks just before and after pilocarpine treatment. EEG monitoring continued for another month after cell ablation. Ablation halted epilepsy progression relative to untreated epileptic mice; the latter showing a significant and dramatic 300% increase in seizure frequency. This increase was prevented in treated mice. Ablation did not, however, cause an immediate reduction in seizures, suggesting that peri-insult generated cells mediate epileptogenesis, but that seizures *per se* are initiated elsewhere in the circuit. These findings demonstrate that targeted ablation of newborn granule cells can produce a striking improvement in disease course, and that the treatment can be effective when applied months after disease onset.

## Introduction

Aberrant integration of newborn hippocampal dentate granule cells is implicated in temporal lobe epileptogenesis. The dendrites and axons of granule cells born in the weeks before and after an epileptogenic injury can develop abnormally, creating recurrent excitatory connections within the dentate gyrus. Cells born after an epileptogenic insult also appear ectopically in the dentate hilus^1–3^. In animal models of epilepsy, these cells are hyperexcitable, exhibiting increased firing rates, depolarized resting membrane potentials, and prolonged action potentials^4,5^. The addition of hyperexcitable newborn neurons is hypothesized to disrupt the “dentate gate”; a proposed function of the healthy dentate that allows it to limit the flow of excitatory signaling through the hippocampus^6^.

Consistent with the hypothesized role of abnormal newborn granule cells in epilepsy, seizure frequency in the pilocarpine model correlates with the percentage of abnormal newborn granule cells^7^, and ablating newborn granule cells or inhibiting neurogenesis *before* the development of epilepsy reduces disease severity^8–11^. The efficacy of ablating newborn granule cells *after* seizure onset, however, had not been assessed. The vast majority of patients with epilepsy present to the clinic after the occurrence of a first seizure, so any broadly useful therapies need to target this population. It is also important to determine whether newborn granule cells still play a role after epilepsy onset, or whether their impact is limited to the prodromal phase of epileptogenesis.

To determine whether eliminating newborn granule cells would be therapeutic in animals with established epilepsy, we used a transgenic mouse model system to express the diphtheria toxin receptor (DTr) in peri-insult generated newborn granule cells. This approach allowed us to ablate these same neurons months after the development of epilepsy by treating the animals with diphtheria toxin (DT).

## Results

**Figure 1:**
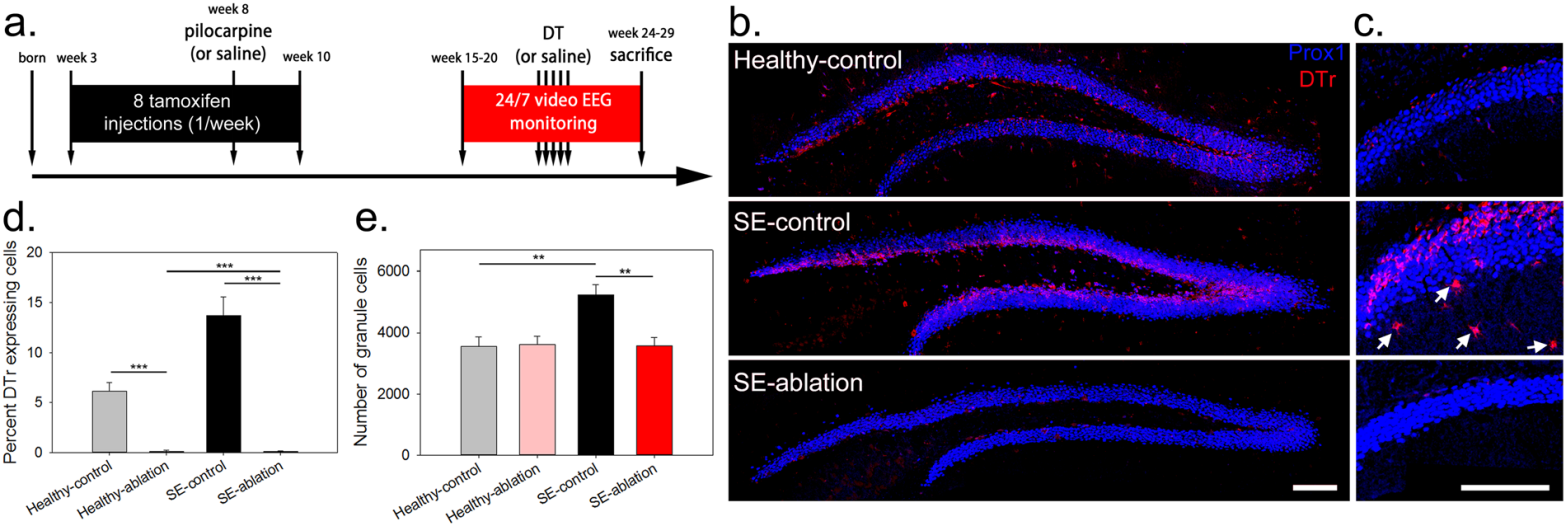
DT ablation effectively eliminates DTr expressing newborn dentate granule cells. (**a**) Timeline depicting the experimental treatment paradigm. (**b**) Images of Prox1 (blue) and DTr (red) immunostained tissue from healthy-control, SE-control and SE-ablation groups. (**c**) Higher resolution images of Prox1 and DTr immunostaining in the dentate gyrus showing DTr induction in a small number of reactive astrocytes in the dentate molecular layer (arrows) of a SE-control mouse. (**d**) Percentage of granule cells that were DTr-positive within each treatment group. (**e**) Number of Prox1-positive dentate granule cells per dentate section. **p < 0.01, ***p < 0.001, scale bars: 100 µm.

Three-week-old NestinCreER^T2^; GFP^+^; DTr^fl/wt^ [DTr-expressing] and NestinCreER^T2^; GFP^+^; DTr^wt/wt^ [DTr-negative] mice were treated with tamoxifen to induce diphtheria toxin receptor (DTr) expression in newborn granule cells. When the mice were eight-weeks-old, they were treated with pilocarpine to induce acute status epilepticus (SE) and the later development of epilepsy. Mice were implanted with cortical electrodes 7–12 weeks after SE, and were monitored by video-EEG 24/7 for one month to establish baseline seizure frequency. Animals then received either diphtheria toxin (DT) or saline, followed by another month of EEG monitoring (Fig. 1). The paradigm produced four treatment groups (Table 1): (1) SE-ablation [epileptic mice with newborn cells ablated], (2) SE-control [epileptic mice with newborn cells intact], (3) Healthy-ablation [non-epileptic mice with newborn cells ablated], and 4) Healthy-control [non-epileptic mice with newborn cells intact].

**Table 1:** Treatment groups.

Group Name	Group	Genotype^	Pilocarpine or control (no SE)	DTr	DT treatment?	Ablation occurred?	Males (n)	Females (n)
Healthy Control	A	NestinCreER^T2^+/*; DTr−/− or NestinCreER^T2^ −/−; DTr+/−	Healthy	DTr negative	DT	NO	2	3
	B	NestinCreER^T2^+/*; DTr+/−	Healthy	DTr positive	Ringer’s	NO	3	2
Healthy Ablation	C	NestinCreER^T2^+/*; DTr+/−	Healthy	DTr positive	DT	YES	5	5
SE Control	D	NestinCreER^T2^+/*; DTr−/− or NestinCreER^T2^ −/−; DTr+/−	Epileptic	DTr negative	DT	NO	3	1
	E	NestinCreER^T2^+/*; DTr+/−	Epileptic	DTr positive	Ringer’s	NO	3	2
SE Ablation	F	NestinCreER^T2^+/*; DTr+/−	Epileptic	DTr positive	DT	YES	4	4

Treatment descriptions and the number of male and female mice present in each animal group. *NestinCreER^T2^ expressing mice are all hemizygous. ^GFP reporter genotype not shown. Tamoxifen treatments (not shown) were identical in all mice. SE, status epilepticus. DTr, diphtheria toxin receptor. DT, diphtheria toxin.

Epileptogenesis significantly increased the number of granule cells (Fig. 1, p = 0.003, two-way ANOVA with Holm-Sidak multiple comparisons procedure [MCP]) and the percentage of DTr-labeled granule cells (Fig. 1, p < 0.001, two-way ANOVA with Holm-Sidak MCP) in SE-control vs. healthy-control mice. This reflects seizure-induced increases in granule cell neurogenesis and survival^12^. DT administration was highly effective, eliminating over 95% of DTr expressing cells (p < 0.001, SE-ablation vs. SE-control) and restoring granule cell numbers to healthy control levels. Interestingly, granule cell numbers were similar in healthy-control and healthy-ablation mice, suggesting either that the majority of cells labeled undergo apoptosis under normal conditions, or that granule cell numbers recover to control values over the one-month period following the last DT injection.

**Figure 2:**
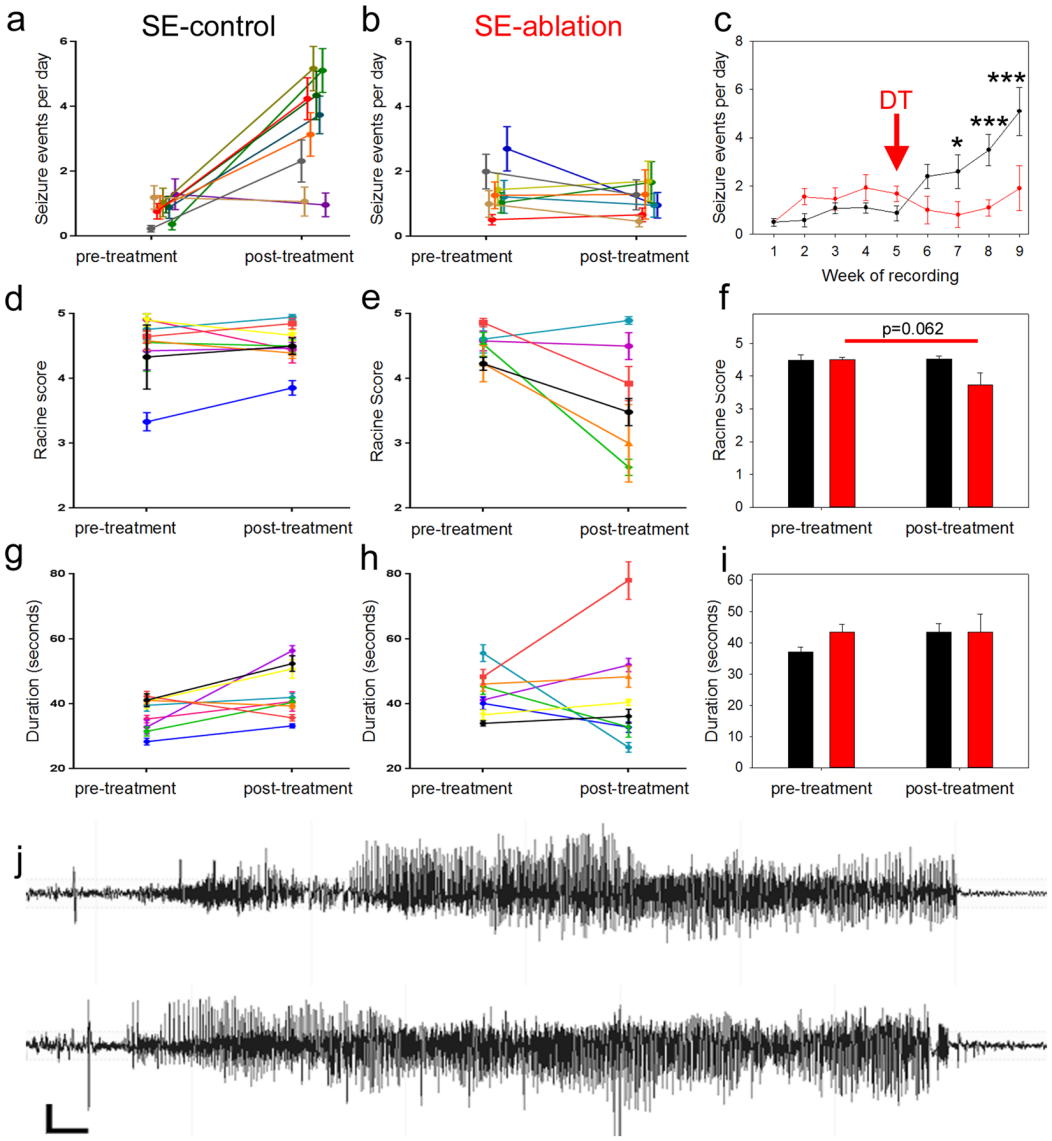
Cell ablation treatment blocks epilepsy progression. Pre-treatment and post-treatment seizure frequencies (**a**,**b**), severities (**d**,**e**), and durations (**g**,**h**) are shown for SE-control mice (left, black) and SE-ablation mice (middle, red). Each line shows the means ± SEM for one animal. (**c**) Average number of seizure events during each week of recording for SE-control (black) and SE-ablation (red) groups (DT was given during week 5, red arrow). (**f**) Average behavioral seizure scores and (**i**) durations. (**j**) Representative post-treatment electrographic seizures from SE-control (top) and SE-ablation (bottom) mice. *p < 0.05, **p < 0.01, ***p < 0.001, scale bars: 300 μV and 2 seconds.

Prior to diphtheria toxin treatment, SE-control and SE-ablation groups exhibited statistically identical seizure frequencies, seizure durations, and behavioral seizure severity scores (Fig. 2; frequency: p = 0.232; severity: p = 0.175; duration: p = 0.379; two-way RM ANOVA with Holm-Sidak MCP). Following treatment, however, disease course in the two groups diverged. In the SE-control group, seizure frequency increased by almost 300% during the post-treatment period (Fig. 2a), reflecting disease progression in these mice (pretreatment: 0.85 ± 0.31 seizures per day; post-treatment: 3.34 ± 0.31 seizures per day; p < 0.001, two-way RM AVOVA with Holm-Sidak MCP). In the SE-ablation group, on the other hand, seizure frequency stabilized (pretreatment: 1.40 ± 0.33 seizures per day; posttreatment: 1.13 ± 0.33 seizures per day; p = 0.561, two-way RM ANOVA with Holm-Sidak MCP). Indeed, four of eight mice exhibited greater than 20% reductions in seizure frequency (Fig. 2b). Behavioral seizure scores^13^ showed a trend towards reduced severity following ablation treatment (Fig. 2d,e,f; p = 0.062, two-way RM ANOVA on ranked data). Seizure duration was unchanged (Fig. 2g,h,i; p = 0.379, two-way RM ANOVA).

A closer analysis of seizure frequency following cell ablation revealed that the positive effects required a few weeks to develop (Fig. 2c; p < 0.001, two-way RM ANOVA). Specifically, differences in seizure frequency were not evident until two weeks after DT treatment (during the seventh week of recording). At this time, SE-control mice exhibited 2.6 ± 0.5 seizure per day, while SE-ablation mice had only 0.9 ± 0.6 seizures per day (p = 0.028, Holm-Sidak MCP). During the final week of recording (week nine) SE-control mice experienced 5.1 ± 0.5 seizures per day and SE-ablation mice exhibited 1.8 ± 0.6 seizures per day (p < 0.001, Holm-Sidak MCP).

### Effect of cell ablation on appearance of hilar ectopic cells

**Figure 3:**
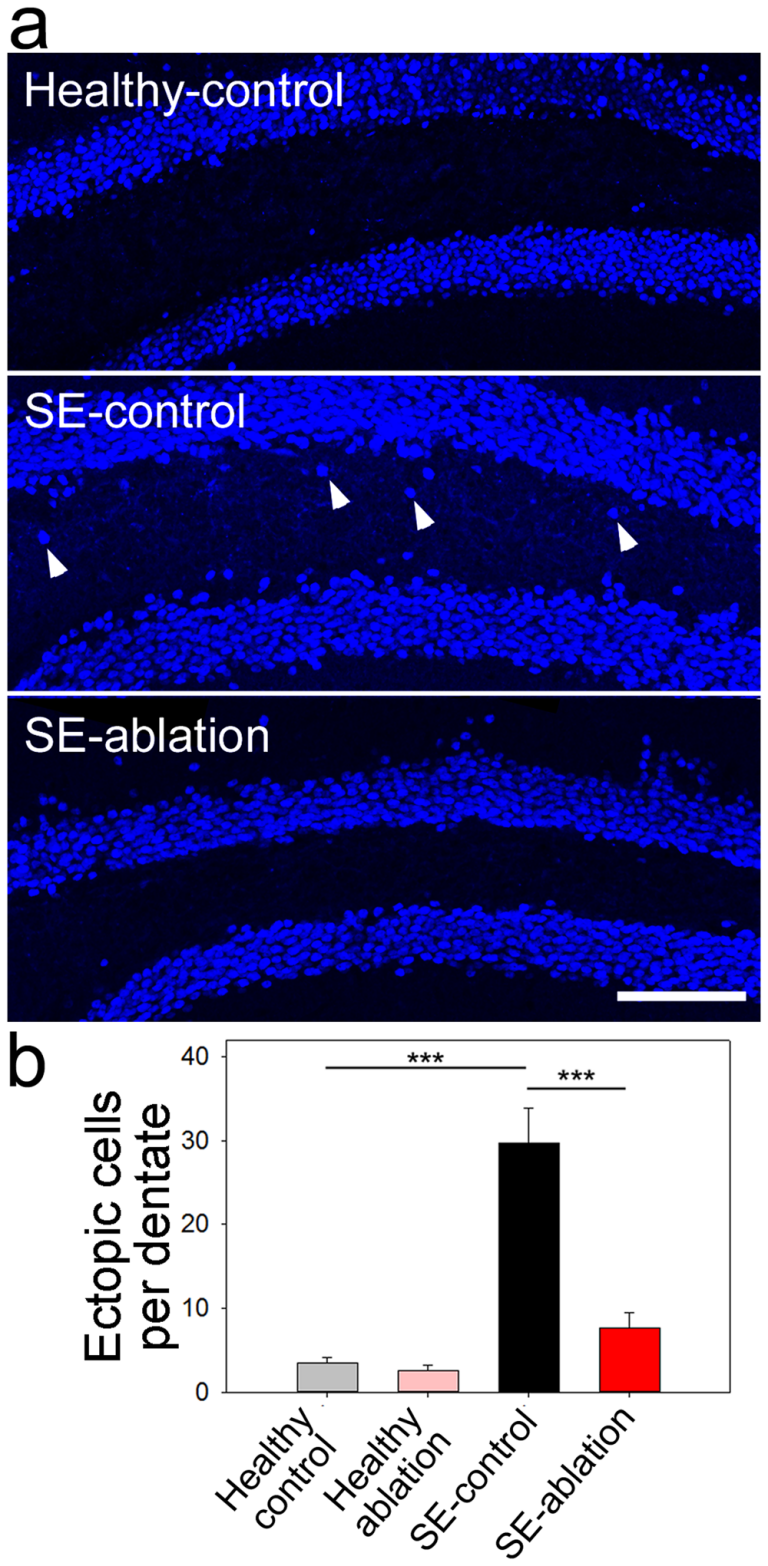
Cell ablation reduces ectopic cell numbers. (**a**) Prox1-stained dentate gyri from healthy-control (top), SE-control (middle), and SE-ablation (bottom) mice. Arrowheads denote ectopic cells. (**b**) Quantification of the number of ectopic granule cells per dentate section in each of the four treatment groups. ***p < 0.001, scale bar: 100 µm.

Hilar ectopic granule cells, which contribute to dentate hyperexcitability^2,4,5,14–17^, were revealed by Prox1 immunostaining. As expected, the number of ectopic granule cells was increased in SE-control mice (Fig. 3a,b; p < 0.001, two-way ANOVA with Holm-Sidak MCP). Cell ablation treatment drastically reduced the number of hilar ectopic cells relative to SE-control mice (p < 0.001), returning numbers to healthy control levels (p = 0.121).

### Effect of cell ablation on mossy fiber sprouting

**Figure 4:**
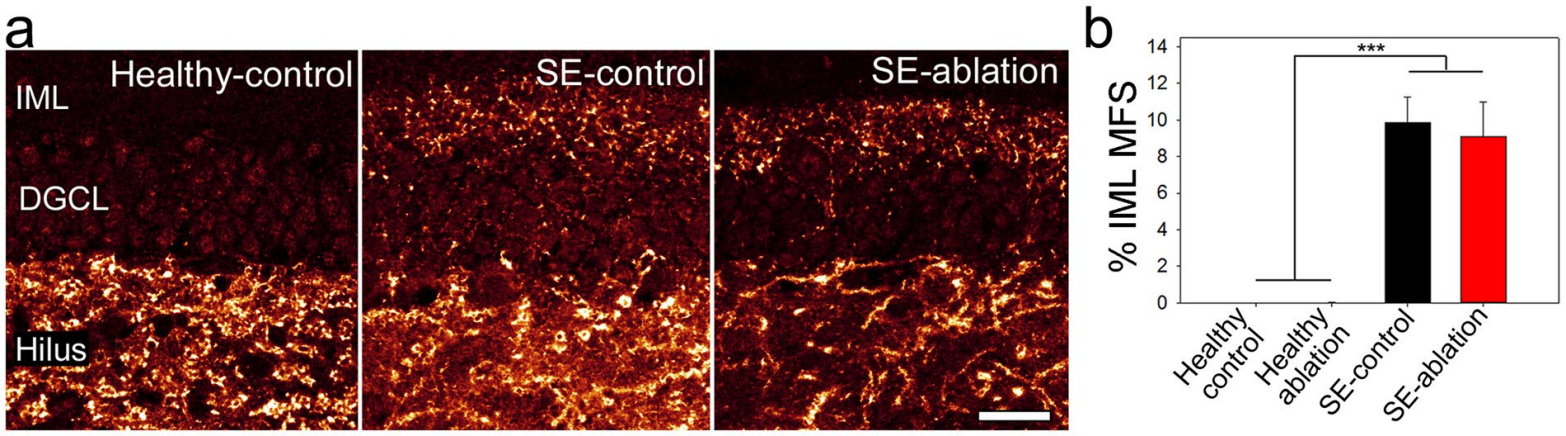
Cell ablation does not reduce mossy fiber sprouting. (**a**) ZnT3-immunostaining of mossy fiber terminals in healthy-control (left), SE-control (middle), and SE-ablation (right) mice. (**b**) Graph shows the percentage of the inner molecular layer (IML) occupied by ZnT3-immunoreative terminals for each group. DGCL = dentate granule cell body layer, ***p < 0.001, scale bar scale bar: 25 µm.

Inner molecular layer mossy fiber sprouting is a prominent feature in many animal models of epilepsy, including the pilocarpine model used here (Fig. 4; epileptic vs. healthy mice, p < 0.001, two-way RM ANOVA). Newborn granule cells contribute to sprouting^18^; therefore we queried whether removing these cells would reduce sprouting. Ablation did not significantly decrease sprouting (p = 0.724), in accord with past studies^10,11^. These findings are consistent with recent data indicating that older cells can contribute to sprouting^19^, perhaps compensating for the loss of younger cells^20^.

### Effect of cell ablation on neurogenesis rates

**Figure 5:**
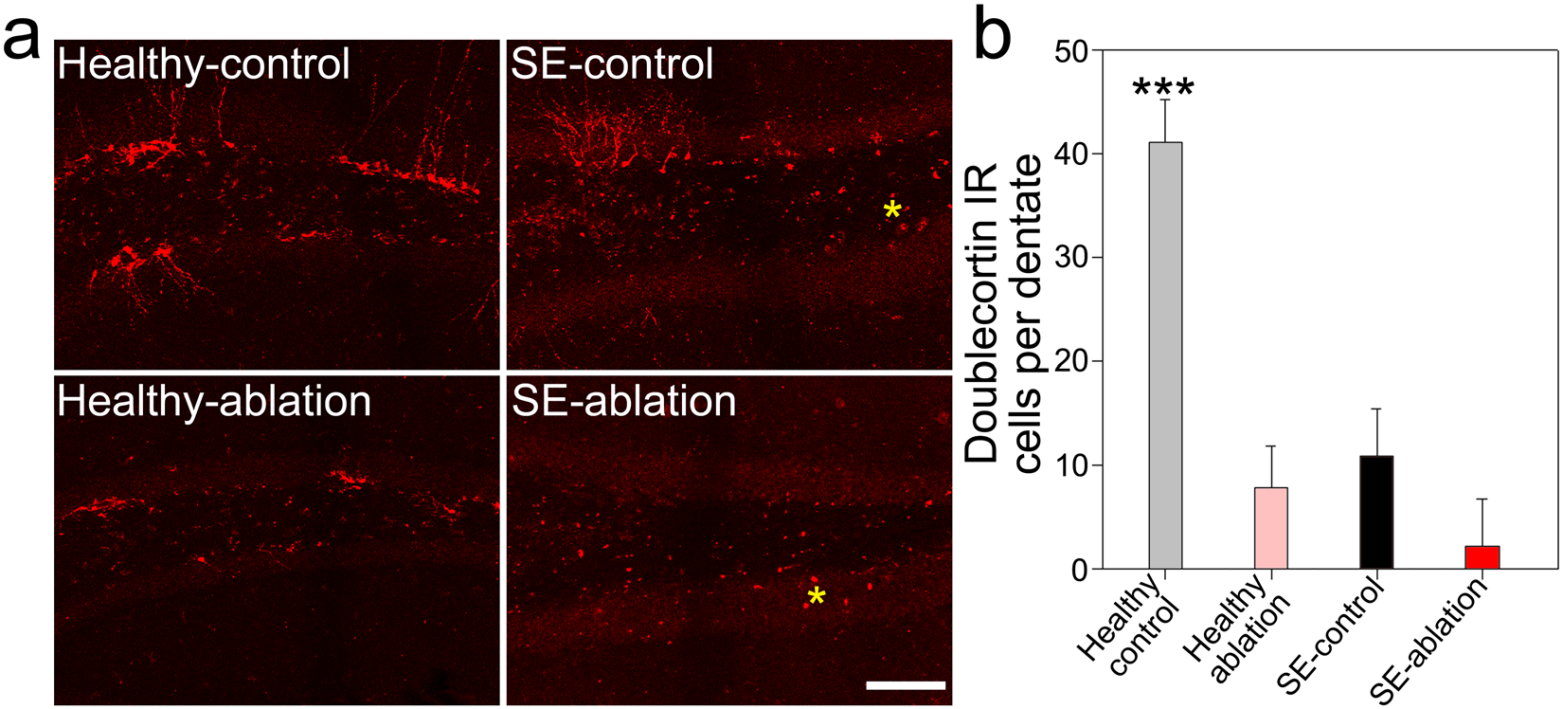
Representative confocal images of doublecortin immunoreactivity in the dentate gyri of (**a**) Healthy-control, healthy-ablation, SE-control and SE-ablation mice. Yellow asterisks denote examples of autofluorescent cellular debris in epileptic mice, and not doublecortin immunoreactivity (**b**) Quantification of doublecortin-immunoreactive granule cells per dentate gyrus in the four groups. ***p < 0.001 vs. all other groups. Scale bar = 100 µm.

To confirm the efficacy of ablation treatment on neurogenesis, brain sections were immunostained with the immature granule cell marker doublecortin. Ablation treatment significantly reduced the number of doublecortin-expressing cells in healthy-ablation mice relative to all other groups (Fig. 5; p < 0.001, two-way ANOVA on ranked data). Interestingly, at the time point examined (4–5 months after status epilepticus), the number of doublecortin-expressing cells per dentate was also reduced in SE-control mice relative to healthy-control mice. Our findings are consistent with numerous studies showing increased neurogenesis in the weeks after status, but impaired neurogenesis in chronically epileptic animals^21–25^. Although doublecortin-expressing cells were numerically fewer in SE-ablation mice relative to SE-control mice, the difference was not statistically significant (p = 0.174). This result almost certainly reflects the chronic timepoint at which the animals were collected, when all SE groups show reduced neurogenesis. Nonetheless, the data clearly indicate that the ablation strategy kills newborn cells. Consistent with this conclusion, in our previous work using the identical genetic strategy, SE-ablation mice had fewer doublecortin-expressing cells than SE-controls three months after status^11^.

### Effect of cell ablation on astrocytes and microglia

The present findings support the conclusion that ablating peri-insult generated newborn granule cells prevents epilepsy progression. Cell ablation, however, has the potential to produce inflammatory changes that might impact seizure occurrence. To assess this possibility, sections were immunostained with the astroglial marker GFAP, and the microglial marker Iba1.

**Figure 6:**
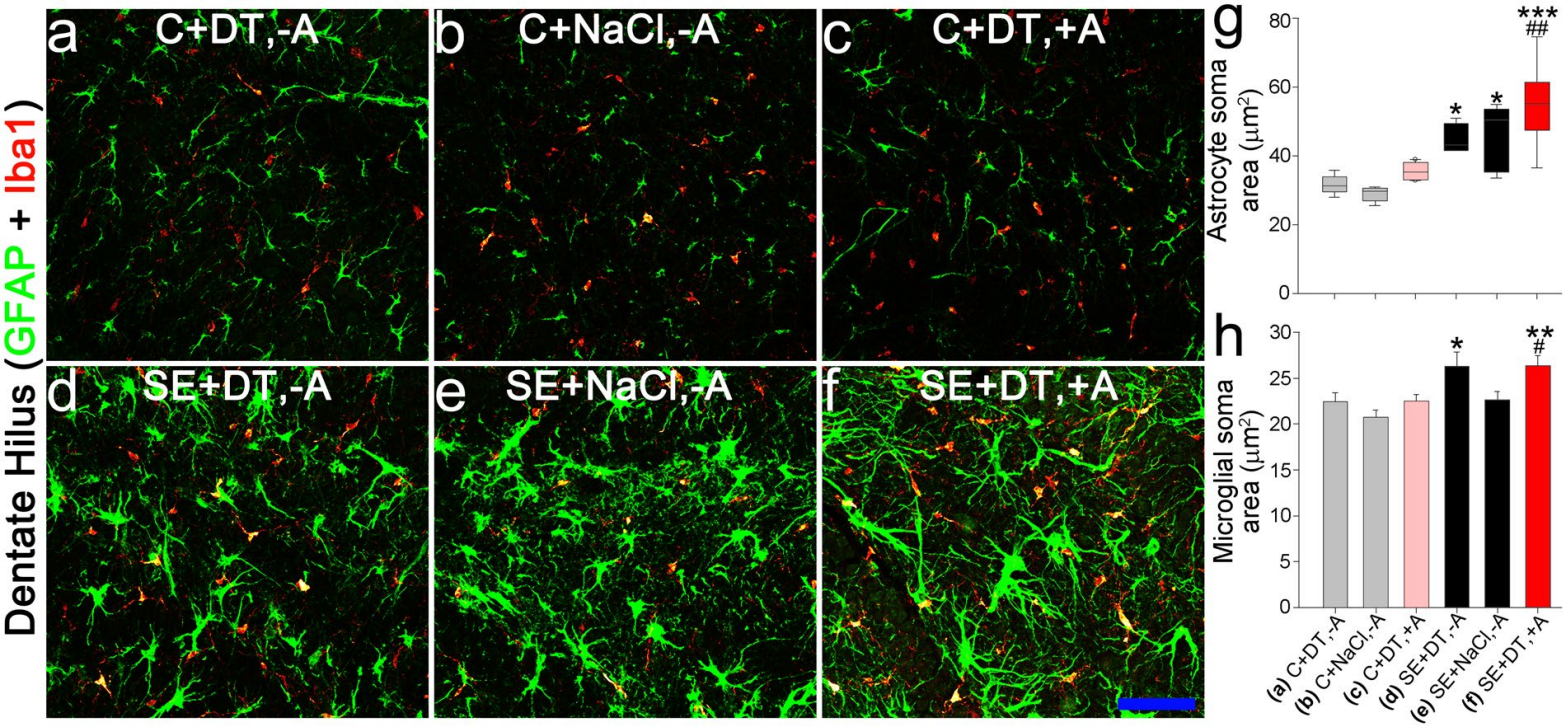
Confocal maximum projections of the dentate hilus showing immunostaining for microglial (Iba1, red) and astrocytic (GFAP, green) markers. (**a**) Healthy-control that received diphtheria toxin but lacked the receptor, so ablation did not occur (C + DT, −A). (**b**) Healthy-control that expressed the diphtheria toxin receptor, but received saline, so ablation did not occur (C + NaCl, −A). (**c**) Healthy-ablation, expressing the receptor and receiving toxin (C + DT, + A). (**d**) SE-control that received toxin but lacked the receptor (SE + DT, −A). (**e**) SE-control that expressed the diphtheria toxin receptor, but received saline (SE + NaCl, −A). (**f**) SE-ablation, expressing the receptor and receiving toxin (SE + DT, + A). Scale bar = 50 µm. (**g**) Graph showing the average soma area for GFAP immunopositive astrocytes. Status epilepticus was associated with increased astrocyte soma area. ***p < 0.001 vs. Group B. *p < 0.05 vs. Group B. ^##^p < 0.01 vs. Group A. (**h**) Graph detailing the average soma area for Iba1 positive microglia cells within each treatment group. Status epilepticus (SE) was associated with increased microglial soma area. **p < 0.01 vs. Group B. *p < 0.05 vs. Group B. ^#^p < 0.05 vs Group C. See Table 1 for group details.

Astrocyte soma area was significantly increased in epileptic mice (Fig. 6; Group F vs. Groups A [p = 0.004; one-way ANOVA on ranks]) and B [p < 0.001]; Group D vs. Group B [p = 0.030] and Group E vs. Group B [p = 0.024]), consistent with prior studies demonstrating that epilepsy is associated with brain inflammation^26–28^. No significant differences were found among epileptic mice. Microglial soma area was also increased in epileptic mice (Fig. 6; Group F vs. Groups B [p = 0.006, one-way ANOVA] and C [p = 0.034]; Group D vs. group B [p = 0.032]). Notably, microglial soma area in epileptic DT-treated mice, in which ablation occurred, was statistically identical to epileptic DT-treated mice without ablation (Group F vs. Group D, p = 0.956, one-way ANOVA), indicating that non-specific DT toxicity cannot account for the seizure-reducing effects of cell ablation.

## Discussion

For the present study, we used a targeted cell ablation strategy to demonstrate that selective removal of peri-insult generated newborn granule cells from the epileptic brain prevents further epilepsy progression. This finding builds upon prior work demonstrating that prophylactic ablation of newborn granule cells reduces seizure incidence once epilepsy develops, and represents the next key step in determining whether targeted cell ablation has potential as a novel therapy for epilepsy. The present findings predict that such a treatment would be beneficial in patients with new onset temporal lobe epilepsy.

### Caveats and limitations

Several studies, using distinct strategies, have now demonstrated that reducing neurogenesis or ablating newborn cells mitigates epilepsy severity^8–11^ (but see also ref.^29^). Together, these findings provide compelling support for the hypothesis that adult neurogenesis contributes to temporal lobe epilepsy. Nonetheless, several caveats should be kept in mind. Firstly, the extent to which newborn cells are critical for epilepsy remains to be determined. In all studies conducted to date, epilepsy still developed or persisted; albeit with reduced severity^8–11^. This may simply reflect the limitations of the approaches used. It has not been possible with current techniques to ablate 100% of newborn cells. Indeed, while our previous work with the identical mouse line and similar treatment strategy confirmed that ablation was effective, we still found only a 50% reduction in doublecortin-expressing newborn granule cells three months after ablation^11^. Remaining cells, therefore, may be sufficient to promote epileptogenesis. Alternatively, newborn cells may modulate epilepsy severity without being required for epilepsy onset. The status epilepticus models used for these studies cause extensive extrahippocampal damage^30^. Seizures might originate from these damaged extrahippocampal regions. In light of this fact, it is somewhat surprising that newborn granule cell ablation works as well as it does. It will be important in future studies to test more focal temporal lobe epilepsy models, where newborn granule cells may play a larger role. A second caveat of the current approach is that surviving progenitor cells may reestablish dentate neurogenesis, potentially limiting the long-term utility of ablation. Because of this limitation, animals were not examined beyond the one month post-ablation period. Whether the positive effects observed here persist beyond this initial period will need to await the development of better techniques for controlling neurogenesis. We do note, however, that Cho and colleagues^10^ demonstrated that ablating granule cell progenitors prior to status epilepticus reduced seizure frequency up to 48 weeks later. A final caveat of the approach is that indirect effects of ablation cannot be entirely excluded. DTr expression was induced in a small number of reactive astrocytes in epileptic mice (Fig. 1), and anti-neurogenic and cell ablation approaches have the potential to alter the hippocampal circuit in unexpected ways (see ref.^31^). It is possible that such off-target effects contribute to the observed reduction in epilepsy severity. The development of new techniques will be required to address these issues. In the meantime, newborn granule cells remain promising candidates for the development of a mechanistic understanding of temporal lobe epileptogenesis.

### Role of newborn granule cells in epileptogenesis

Epilepsy encompasses multiple processes which may or may not reflect distinct underlying mechanisms. Epilepsy begins with epileptogenesis, which includes 1) the transition from a brain that does not support spontaneous seizures to a brain that does and 2) increases in disease severity that can occur after clinical disease onset, also known as epilepsy progression^32^. Once the epileptic state is established, neurons and/or neuronal circuits in the brain occasionally initiate seizures. Whether the neurons responsible for epileptogenesis are the same neurons that initiate seizures is not known.

While previous studies have consistently found that reducing the number of newborn granule cells prior to disease onset mitigates the severity of the epilepsy that subsequently develops, these studies provide little insight into whether the newborn cells play a role in epilepsy progression, seizure initiation, or both. Either scenario could account for the observed results. By contrast, the present findings argue for a role for newborn cells in epilepsy progression, but not seizure initiation. Specifically, ablation of peri-insult generated newborn cells after the onset of epilepsy produced no acute change in seizure frequency. A significant difference in seizure frequency was not evident until two weeks after ablation, and in this case only because seizure frequency increased in untreated animals; not because ablation-treated animals experienced reductions (see Fig. 2c). If newborn granule cells were responsible for seizure initiation, then eliminating these cells would be predicted to produce an immediate reduction in seizure frequency, on par with the reduction observed in our prior work. Rather, the findings suggest that these newborn cells play a role in epilepsy progression, and that seizure initiation occurs elsewhere in the circuit. If correct, such a model would be reminiscent of memory consolidation, in which hippocampal function is only required during early phases of memory retention^33,34^.

### Significance of the findings for epilepsy therapy

The present findings suggest it may be possible to overcome one of the major limitations of epilepsy therapy development. Specifically, the vast majority of interventions that have shown disease modifying effects in animal models of epilepsy began treatment either before, or immediately after, an epileptogenic insult. This greatly complicates translating these approaches to clinical populations because (1) many patients develop epilepsy in the absence of an identifiable initial insult and (2) it has so far not been possible to predict which patients will develop epilepsy even when the causal insult (e.g. brain trauma) is known. Deciding who to treat, therefore, is extremely challenging; particularly if therapies have significant side effects. The efficacy of the approach used here provides hope that the window of opportunity for disease modifying treatments in epilepsy extends beyond the first clinical seizure, when patients could be easily identified.

One caveat to our approach is that we targeted cells born before and after the insult; the peri-insult generated population of granule cells. The rationale for targeting granule cells born before the insult is based on numerous studies demonstrating that these neurons contribute to hippocampal rewiring (for review see ref.^24,35^). Similarly, granule cells born after the insult also integrate abnormally. If the goal is to remove abnormal granule cells from the dentate, therefore, both populations must be targeted. A challenge moving forward is developing strategies that could achieve this in patient populations. While the transgenic strategy used here is not translatable, it should be possible to eliminate the same population of cells in patients by taking advantage of proteins expressed exclusively in these cells, like doublecortin, for targeting. The window of time during which such an approach would be viable is still narrow, as developing neurons down regulate the known selective markers as they mature. A promising strategy for chronic epilepsy would be to identify differences in gene expression between normal and abnormal granule cells. Although an optimal target has yet to emerge, established differences in morphology and physiology indicate that these cells likely exhibit differences in growth-associated proteins, synaptic components and ion channels^5,14,15^. With additional research, it may be possible to selectively target abnormal cells for modulation or elimination as a novel therapy for epilepsy.

## Methods

### Animals

All procedures complied with the National Institutes of Health’s and institutional guidelines for the care and use of animals and have been approved by CCHMC’s Institutional Animal Care and Use Committee (IACUC). Mice used in this study were derived from NestinCreER^T2^ mice^36^, Gt(ROSA)26Sor^tm1(HBEGF)Awai^/J mice^37^ (referred to as DTr mice), and GFP reporter mice^38,39^. NestinCreER^T2^ mice were acquired from Dr. Lionel Chow (CCHMC) and were maintained on an FVB/NJ background. Diphtheria toxin-receptor (DTr) mice and GFP mice were maintained on a C57BL/6 background. All mice in the study were the F1 progeny of hemizygous NestinCreER^T2^ mice (FVB/NJ) crossed to DTr^+/−^; GFP^+/±^ expressing mice (C57BL/6). All mice, therefore, were a 50:50 mix of FVB/NJ and C57BL/6 backgrounds. This cross was used to generate animals of multiple genotypes, as outlined in Table 1. Mice were housed in standard cages with regular bedding within the CCHMC clean barrier vivarium facility and were provided with regular chow and water *ad libitum* on a 14/10 day/night cycle. Mice were weaned between P21-P23 and same-sex littermates were housed together with 2–4 mice per cage.

### Experimental Design

A total of 146 transgenic mice were generated for potential use in the present study. One hundred twenty-three mice were designated for pilocarpine treatment (SE) and 23 for saline treatment (healthy). Eighty mice successfully entered and survived status epileptics (65%). From this group of 80 mice, 39 were assigned to EEG monitoring. Groups were chosen at random immediately following pilocarpine treatment by the researcher (BEH). EEG recording platforms are limited such that no more than 16 mice can be monitored at any given time, and space is shared between multiple studies in multiple labs, so not all mice could be used. An additional 22 SE mice were excluded from the final group due to surgical/electrode failures (n = 11), early mortality (n = 9), or an absence of spontaneous seizures during baseline recording (n = 2). Of the 23 healthy control mice, three were excluded due to surgical/electrode failures or early mortality. The final study included 17 pilocarpine-treated mice, and 20 healthy controls. Mice were randomly assorted into the four principal groups used in this study: (1) Healthy-control (n = 10), (2) Healthy-ablation (n = 10), (3) SE-control (n = 9) and (4) SE-ablation (n = 8). Using data generated from prior studies, we calculated that we should be able to detect a 50% change in seizure frequency with 95% confidence and a power > 0.8 with group sizes of 8 mice (0.461 seizures/day, SD 0.406, n = 10) in all epileptic groups. Blind analyses were conducted on data collected from final group constituents by concealing and coding samples prior to analysis.

### Tamoxifen and pilocarpine

All mice received eight, once-weekly subcutaneous injections of tamoxifen (Sigma, 250 mg/kg/dose at a concentration of 20 mg/mL in corn oil) beginning the day they were weaned (P21-P23). Mice underwent pilocarpine-induced SE at eight weeks of age, such that five tamoxifen injections occurred before status, and three after. SE was induced in an empty, bedding-free cage. Mice received an injection of methyl scopolamine nitrate (1 mg/kg intraperitoneally dissolved in sterile Ringer’s solution). Fifteen minutes later, mice received an injection of pilocarpine (380 mg/kg intraperitoneally dissolved in sterile Ringer’s solution [Table 1, groups D-F]; no SE controls received sterile Ringer’s [groups A-C]). Immediately following pilocarpine administration, animal behavior was monitored continuously for seizure activity. Onset of SE was defined by the appearance of multiple class V (tonic/clonic) seizures^13^, followed by continuous behavioral seizure activity. In the event an animal did not develop SE within 60 minutes following pilocarpine administration, a second injection of pilocarpine (190 mg/kg) was administered. Three hours after the onset of SE mice received two injections of diazepam spaced 15 minutes apart (10 mg/kg subcutaneously). Mice were then returned to normal housing conditions. Animal health was monitored closely in the days following SE. Sterile Ringer’s solution was provided s.c. as needed to restore mice to pretreatment weight. Following pilocarpine (or control) treatment mice were assigned to one of six treatment groups, as shown in Table 1.

### EEG monitoring and DT administration

Seven to twelve weeks following SE mice underwent electrode implantation surgery^40^. Three mice that did not receive pilocarpine were also implanted for EEG monitoring. Seizures were not observed in any mice that were not previously treated with pilocarpine. Mice were anesthetized with 4.0% isoflurane in 1.5% oxygen, transferred to a stereotaxic frame, and kept sedated with 0.5–1.0% isoflurane. The surgical site was shaved and cleaned with Dermachlor (2.0% chlorhexidine) and 70% ethanol. Lidocaine (50 μL) was administered subcutaneously at the surgical site. A small incision was made above the skull and two burr holes were drilled through the skull, but leaving the dura intact. Holes for electrodes were placed at the following coordinates: 1.5 mm anterior of lambda and 1.5 mm left and right of the sagittal suture. Three additional holes were placed for skull screws (two at the base of the skull, 1.5 mm posterior to lambda and 1.5 mm left and right of center, and the third near the front of the skull, 1.5 mm posterior to bregma and 2.0 mm right of the sagittal suture). The grounding and recording electrodes from a single channel wireless EEG transmitter (TA11ETA-F10, Data Sciences International) were placed beneath the skull and above the dura, and the transmitter body was inserted into a pocket created underneath the skin of the animals’ torso. Dental cement was applied to secure the transmitter leads and the surgical wound was sutured. Mice were housed in single cages with standard bedding and food and water *ad libitum*. Cages were placed on wireless receiver plates (RPC1, Data Sciences International) and continuous video-EEG monitoring was initiated. Animal behavior was monitored to ensure a complete recovery. Four weeks into the recording period mice received five, once-daily injections of diphtheria toxin (DT). DT was dissolved in nuclease-free sterile water and injected intraperitoneally at a dose range of 30–50 μg/kg. DT potency was found to vary among lots, so effective doses were established empirically (data not shown). Final DT doses were statistically equivalent among epileptic animals (p = 0.368, Mann-Whitney rank sum test). A subset of control mice received sterile Ringer’s solution rather than DT. Following DT treatment, mice were video-EEG monitored for an additional three to four weeks.

### EEG analysis

Video-EEG data was analyzed by a reviewer blind to treatment group using Neuroscore software (version 2.1.0). EEG data was analyzed for seizure frequency, severity, and duration. A seizure was identified by a sudden increase in voltage amplitude (at least 2x baseline), with a progressive change in firing frequency or amplitude, and a minimum duration of 10 seconds. Seizure cessation was marked when the recording returned to baseline, although postictal theta was not included as part of the seizure for duration measurements. Behavioral seizure severity was determined by video analysis of each electrographic seizure using the Racine scale^13^. Seizure severity was only scored when there was a clear video image of the mouse experiencing the electrographic seizure.

### Tissue processing

At the end of the EEG recording period, mice were anesthetized with an overdose of pentobarbital (100 mg/kg). Mice were transcardially perfused with heparinized PBS (1 U/mL) followed by 2.5% PFA with 4.0% sucrose in PBS chilled on ice. Brains were dissected and fixed overnight in 2.5% PFA with 4.0% sucrose in PBS at 4 °C. Brains were cryoprotected through an ascending sucrose series (10%, 20%, 30% in PBS at 4 °C) for a minimum of 24 hours per step. Brains were snap frozen in 4-methylbutane chilled to −25 °C and stored at −80 °C until further use. Brains were sectioned coronally to a thickness of 60 μm using a cryostat cooled to −20 °C. Sections were left to dry at room temperature overnight and stored at −80 °C until used for immunohistochemistry. Sections were thawed in PBS and stained with the following primary antibodies: goat anti-HBEGF (to stain DTr, R & D Systems, 1:150), rabbit anti-Prox1 (Sigma, 1:1000), chicken anti-GFP (Abcam, 1:500), rabbit anti-ZnT3 (Synaptic Systems, 1:3000), chicken anti-GFAP (Millipore, 1:500), goat anti-doublecortin (Santa Cruz 1:100), and rabbit anti-Iba1 (Synaptic Systems, 1:1000). The following secondary antibodies were used: donkey anti-goat 594, donkey anti-rabbit 647, goat anti-chicken 488, and goat anti-rabbit 594 (all from Life Technologies, 1:750). Immunostained sections were dehydrated in serial ethanol washes (50%, 70%, 95%, 100%, 100% in diH_2_O), cleared in xylenes, and hard mounted with Krystalon. All sections were coded prior to confocal imaging so that the researcher was blind to treatment group during data collection and analysis.

### Quantification of doublecortin-expressing, and DTr + Prox1-expressing granule cells

Sections immunostained for doublecortin or double-immunostained for Prox1 + DTr were imaged using a 3024 Nikon A1Rsi inverted microscope with a 60x water objective (NA = 1.27, resolution = 0.410 μm/pixel). Confocal image “stacks” through the z-depth were collected at 1 μm increments through 20 μm of tissue. Multiple image stacks were montaged to capture the entire x-y dimensions of the dentate gyrus (including the hilus) from two hemispheres (left and right) per mouse. Data collected from each hemisphere was averaged for each animal prior to statistical analysis. Images of Prox1 + DTr immunostaining were collected simultaneously.

To determine the number of doublecortin-expressing and Prox1 + DTr-expressing granule cells present within a 20 μm thick section of the dentate gyrus, confocal image stacks were imported into Imaris software (version 7.7.2). Immunopostive cells were quantified using an automated detection method which identifies and counts fluorescent “spots”. Minimum fluorescent diameter was set to 5.0 μm and minimum intensity threshold was adjusted to optimize cell detection. All counts were completed using an optical dissector approach, excluding all cell bodies truncated at the upper surface of the tissue to eliminate bias due to changes in cell size or shape^41,42^. The automated cell counts were then reviewed to remove false positives and identify false negatives. Counts are expressed as number of granule cells per dentate gyrus section (encompassing the entire x-y dimensions of a dentate section and 20 µm through the z-depth).

The location of each identified dentate granule cell was subsequently analyzed to determine the number of hilar ectopic granule cells present in each dentate section. Prox1-immunoreactive cells located within the hilus that were a minimum of 20 μm away from the hilar-granule cell body layer border were considered ectopic.

To determine the percentage of Prox1 immunoreactive granule cells which were co-labeled with DTr, images of Prox1 + DTr immunostained sections were cropped in the x and y dimensions to isolate a 400 µm section of the dentate at the midpoint of the upper blade. Prox1 + cells within these samples were then identified and assessed for coexpression of DTr. Percent DTr expression among granule cells was determined using the following formula (DTr and Prox1 coexpressing cells/all Prox1 expressing cells) × 100.

### Mossy fiber sprouting

Sections immunostained for GFP + ZnT3 (approximately 2.4 mm posterior to bregma) were used to quantify mossy fiber sprouting^43^. Sections were imaged using a DMI6000 Leica SP5 inverted microscope with a 63x oil objective (numerical aperture = 1.4). Single images of ZnT3 staining were collected from the midpoint of the upper and lower blades of two hemispheres (resolution = 0.242 µm/pixel). Each image was collected 2–3 μm beneath the surface of the tissue to control for antibody penetration. Images were imported into Neurolucida software (version 11.09) for analysis. ZnT3 immunoreactivity within the inner molecular layer was determined using an automated object detection analysis. Detection parameters were set to an intensity threshold determined by the reviewer to optimize puncta identification. Objects less than 0.5 μm in diameter, the minimum size of granule cell mossy fiber puncta, were excluded. The automated detection was then reviewed by a blinded investigator to identify false negatives and remove false positives. Percent mossy fiber sprouting was calculated as follows: (area of inner molecular layer ZnT3 immunoreactivity/area of inner molecular layer examined) × 100. Percent mossy fiber sprouting from the upper and lower blades was averaged for each animal prior to statistical analysis.

### Astrocyte and microglia soma area measurements

Sections immunostained for GFAP + Iba1 (approximately 2.7 mm posterior to bregma) were imaged using the Leica SP5 system. The soma areas of GFAP- and Iba1-immunostained cells were assessed from confocal image stacks collected from a sample of the dentate hilus from the left and right hemispheres of one section per mouse (20 µm depth, 1 μm step, resolution = 0.484 μm/pixel) using Neurolucida software. Ten GFAP-immunoreactive and ten Iba1-immunoreactive cells per hemisphere were randomly selected for quantification (for a total of 20 cells of each type per animal). Only somas entirely contained within the image stack were selected for analysis. Maximum soma profile area was determined for each cell. Area measurements within each cell type were averaged for each animal prior to statistical analysis.

### Statistical analysis

Statistical analyses were conducted using Sigma Plot software (version 13.0). Values presented are least square means ± standard error of the mean (SEM), or means ± SEM. No statistical differences were found between male and female mice for any of the parameters presented (Student’s t-test, data not shown), so data were binned. No significant differences between healthy control (non-epileptic), DTr-negative mice receiving DT (Table 1, Group A) and healthy control, DTr-positive mice receiving Ringer’s (Group B) were found for the following measures using Student’s t-test (data not shown): number of granule cells per dentate, number of ectopic cells per dentate, percentage of ectopically located granule cells and percent mossy fiber sprouting, so data were binned. Similarly, no differences between pilocarpine-treated, DTr-negative mice receiving DT (Group D) and pilocarpine-treated, DTr-positive mice receiving Ringer’s (Group E) were found for seizure frequency, seizure severity, seizure duration, number of granule cells per dentate, number of ectopic cells per dentate, percentage of ectopically located granule cells and percent mossy fiber sprouting, so data were also binned. Statistical differences among groups were observed for microglial and astrocyte soma area, so data for all six groups are presented. Primary measures in the study, however, used binned datasets to generate the following groups for statistical analysis: 1) Healthy-control, 2) Healthy-ablation, 3) SE-control and 4) SE-ablation. Group details are presented in Table 1.

EEG data was segregated into “pre-treatment” and “post-treatment” periods. Pre-treatment encompassed the time period up to the first DT injection, while the post-treatment period began seven days after the first DT injection. The DT-treatment period, during which cell death is occurring, was excluded from most analyses; although this data is shown in Fig. 2c. Two SE-ablation mice did not exhibit any seizures in the post-treatment recording period that coincided with a clear video image, therefore they were not included in the behavioral seizure analysis and the subsequent data was generated from six SE-ablation mice and nine SE-control mice. SE-ablation mice were recorded for an average of 26.3 ± 2.7 days during the pre-treatment period and 25.3 ± 4.0 days during the post-treatment period. SE-control mice were recorded for 27.4 ± 2.8 pre-treatment days and 27.7 ± 2.3 post-treatment days (recording times did not differ between groups; pretreatment p = 0.386; post treatment p = 0.143, t-test). SE-ablation mice were implanted on average 74.5 ± 10.7 days following SE, while SE-control mice were implanted 83.0 ± 10.4 following SE (p = 0.118, t-test). SE-ablation mice were sacrificed 136.0 ± 20.4 days following SE, and SE-control mice were sacrificed 149.6 ± 12.4 days following SE (p = 0.114, t-test).

### Figure preparation

All images were prepared using Adobe Photoshop Elements 12. Brightness and contrast were adjusted to optimize cellular detail. Identical changes were made to figures meant for comparison. Graphs were generated using Sigma Plot software (version 13.0).

### Data availability

The datasets generated and/or analyzed for the current study are available from the corresponding author upon reasonable request.

### Significance Statement

Here, we demonstrate that targeted ablation of peri-insult generated granule cells has anti-epileptogenic effects when applied months after disease onset. Our findings provide compelling new evidence that abnormal granule cells play a fundamental and protracted role in the epileptogenic process, rather than a transient and more limited role in modulating the acute effects of the initial injury. Our findings also represent the first proof-of-concept demonstration that cell ablation could be therapeutic in patients who have already developed the disease.
